# Tumor diversity and evolution revealed through RADseq

**DOI:** 10.18632/oncotarget.18355

**Published:** 2017-06-03

**Authors:** Elizabeth B. Perry, Alvin Makohon-Moore, Caihong Zheng, Charles K. Kaufman, Jun Cai, Christine A. Iacobuzio-Donahue, Richard M. White

**Affiliations:** ^1^ Cancer Biology & Genetics, Memorial Sloan Kettering Cancer Center, New York, New York, USA; ^2^ Biostatistics, Yale University, New Haven, Connecticut, USA; ^3^ The David M. Rubenstein Center for Pancreatic Cancer Research, Human Oncology and Pathogenesis Program, Memorial Sloan Kettering Cancer Center, New York, New York, USA; ^4^ Key Laboratory of Genomic and Precision Medicine, Beijing Institute of Genomics, Chinese Academy of Sciences, University of Chinese Academy of Sciences, Beijing, China; ^5^ Washington University School of Medicine, St. Louis, Missouri, USA

**Keywords:** restriction-site associated DNA sequencing, RADseq, cancer, next-generation sequencing, tumor evolution

## Abstract

Cancer is an evolutionary disease, and there is increasing interest in applying tools from evolutionary biology to understand cancer progression. Restriction-site associated DNA sequencing (RADseq) was developed for the field of evolutionary genetics to study adaptation and identify evolutionary relationships among populations. Here we apply RADseq to study tumor evolution, which allows for unbiased sampling of any desired frequency of the genome, overcoming the selection bias and cost limitations inherent to exome or whole-genome sequencing. We apply RADseq to both human pancreatic cancer and zebrafish melanoma samples. Using either a low-frequency (SbfI, 0.4% of the genome) or high-frequency (NsiI, 6-9% of the genome) cutter, we successfully identify single nucleotide substitutions and copy number alterations in tumors, which can be augmented by performing RADseq on sublineages within the tumor. We are able to infer phylogenetic relationships between primary tumors and metastases. These same methods can be used to identify somatic mosaicism in seemingly normal, non-cancerous tissues. Evolutionary studies of cancer that focus on rates of tumor evolution and evolutionary relationships among tumor lineages will benefit from the flexibility and efficiency of restriction-site associated DNA sequencing.

## INTRODUCTION

The characterization of cancer as an evolutionary process was reviewed by Peter Nowell four decades ago. He hypothesized a stepwise progression of acquired variation and natural selection to explain the emergence and increasing aggressiveness of tumors [1]. Since then, data from cancer genomics, multi-region sequencing, and single-cell sequencing have provided new insight into the complex clonal evolution of tumors and the extensive genetic heterogeneity present within and between cancer patients. The mechanisms underlying the generation and maintenance of tumor diversity are of particular interest to those seeking to understand disease progression and therapeutic resistance of advanced cancers. A multidisciplinary approach has been increasingly applied to problems of clonal evolution in cancer [2–6]. Theory and tools from evolutionary biology have been applied to the field of cancer biology to understand the rates of accumulation of mutations in cellular lineages [5, 7], spatial and temporal patterns of intratumor heterogeneity [8–10], the timing and order of metastatic progression [11–14], and optimal strategies for therapeutic dosing and schedules [15–17]. However, a large disciplinary divide still exists between cancer biology and evolutionary biology, and potentially useful theoretical and experimental tools have yet to be applied across disciplines.

**Figure 1:**
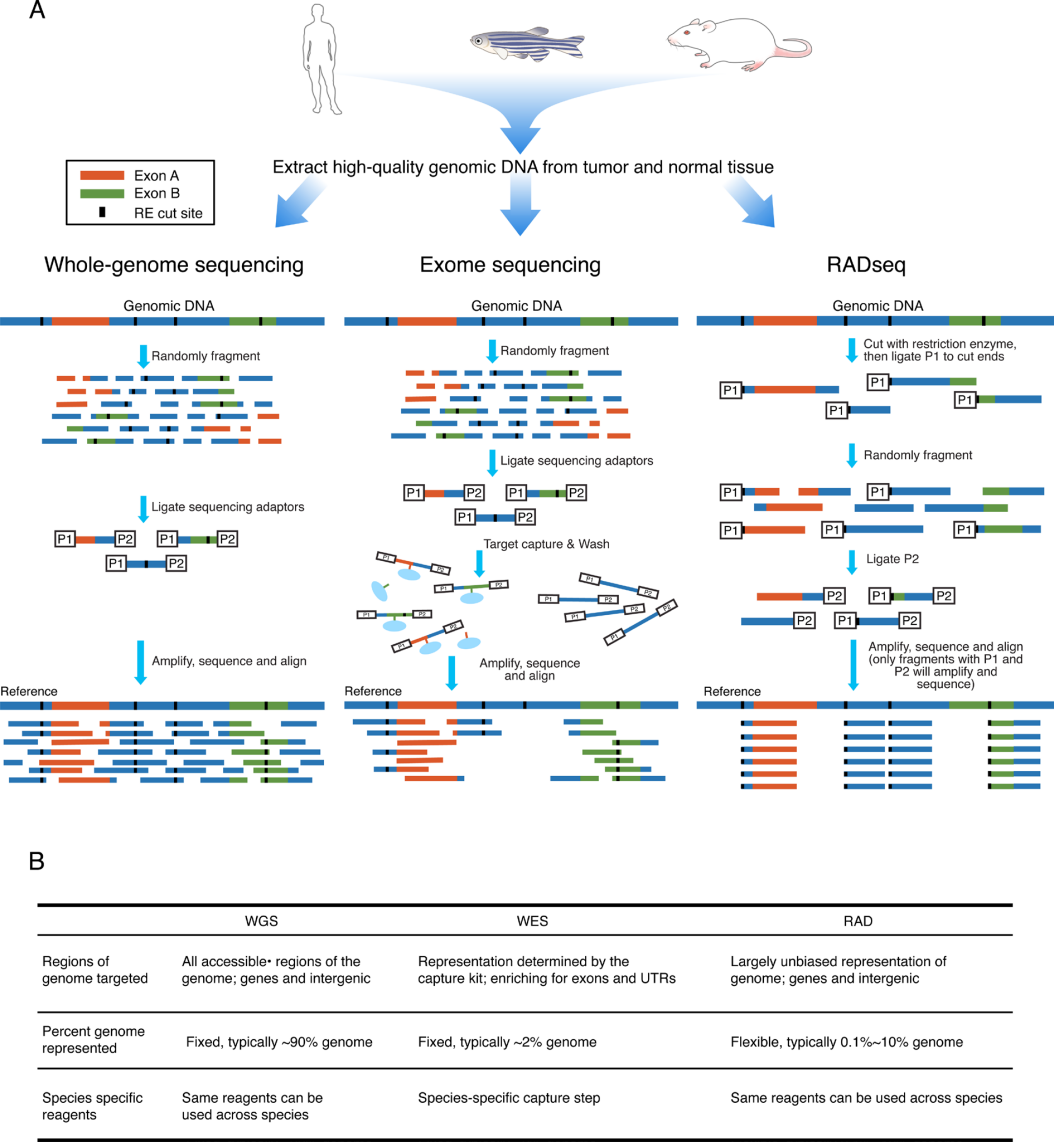
**A. Comparison of whole-genome, exome and RADseq approaches to cancer genome**. Whereas whole genome sequencing allows for an unbiased view of the genome, it requires high cost when high sequencing depth is needed. Exome sequencing is a reduced representation approach that is cost-effective but gives a highly biased view of only protein coding genes, which are under selection in many cancers. RADseq uses restriction sites naturally dispersed across the genome at both intergenic as well as coding regions, combining the benefits of whole-genome and exome sequencing to allow for unbiased, high sequencing depth in a highly cost effective manner. **B**. Specific features of the 3 methods as they apply to cancer biology.

Restriction-site associated DNA sequencing (RADSeq) was initially developed for studies of evolutionary genetics and has proven to be a powerful tool in this field. This method has been largely overlooked in cancer biology, which instead relies almost exclusively on whole-genome sequencing and exome sequencing to discover and quantify genetic variation in tumors (see Figure 1 for a comparison of the three methods). Like exome sequencing, RADSeq is a reduced-representation sequencing approach that targets a subset of the genome. However, instead of targeting exonic regions for sequencing, the RADseq protocol targets regions of the genome flanking restriction enzyme cut sites. Several variations of RADseq have been described [18–24]. In brief, high quality genomic DNA is first digested with a restriction enzyme. Then, sequencing adapters (double-stranded oligos compatible with a next generation sequencing platform) are ligated onto the characteristic sticky ends generated by the restriction enzyme digestion. The highly-specific ligation of sequencing adapters to digested cut sites allows for the targeted sequencing of regions flanking these positions, therefore no capture step is required. Barcode sequences can also be included in the adapter oligos to allow for multiplexed sequencing of multiple samples in the same sequencing lane.

RADseq is a highly flexible approach because the proportion of the genome targeted for sequencing can be controlled through the choice of restriction enzyme. Common cutters (typically enzymes with a shorter recognition sequence,) will target a higher percentage of genome for sequencing than rare cutters. This means that common cutters can be used for questions that require more sequence information per genome, for example to distinguish between recently-diverged populations (e.g. primary tumors *vs*. metastases) or tumors with low mutation rates/burdens (e.g. pediatric tumors). Rare cutters can be selected for research questions that call for fewer sites per genome and a greater depth of sequencing per site (e.g. heterogeneity questions), or benefit from a large number of samples (e.g. many tumor sites or patients).

RADseq differs from whole-exome sequencing in that it does not specifically target functional regions of the genome. Restriction cut sites occur throughout the genome, largely without bias, and thus the sequenced regions will represent coding DNA as well as many intergenic and other non-coding sites where non-coding RNAs might be transcribed as well. This important distinction means that RADseq will likely not be the most efficient method to identify driver mutations and protein changes responsible for the cancer phenotype. However, RADseq does have the potential to be a more useful tool than exome sequencing to study tumor phylogenetics and intrinsic mutation rates, for which neutrally evolving sites provide the most robust information [25–27]. These differing strengths make whole-exome sequencing and RADseq complimentary methods for a variety of cancer biology questions. Here we test the utility of RADseq for cancer genomics by applying RADseq to a zebrafish model of melanoma and to human pancreatic tumors.

## RESULTS

### Transgenic zebrafish model of melanoma

#### Performance

**Figure 2:**
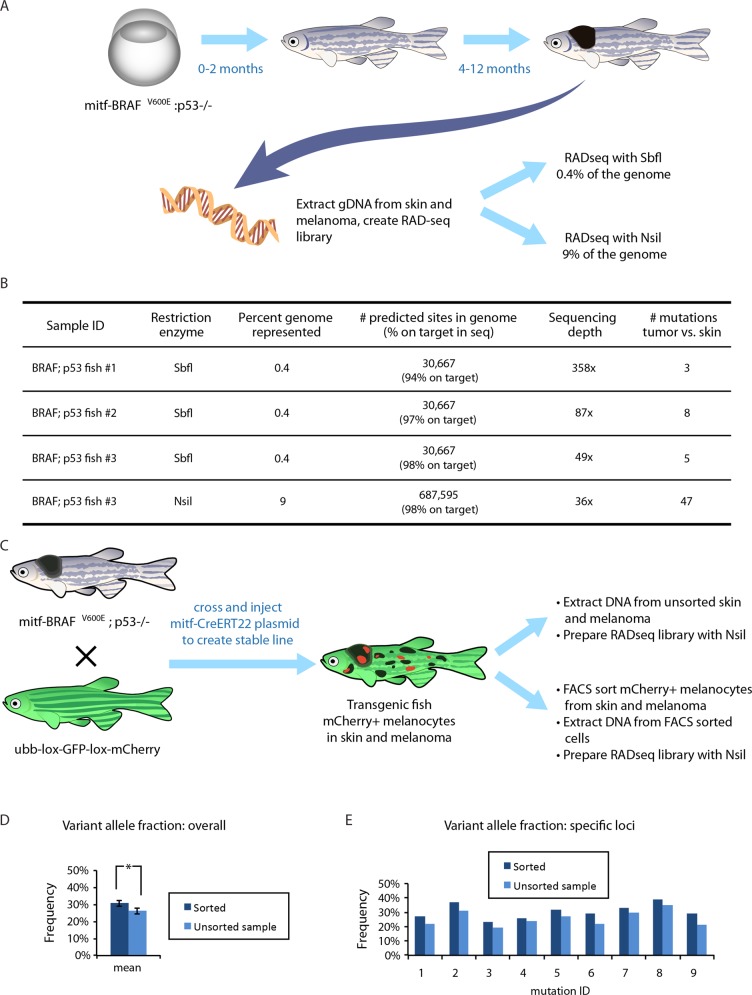
Applying RADseq to a zebrafish model of melanoma **A**. A transgenic zebrafish model in which the mitfa promoter drives human oncogenic BRAFV600E. The embryos (left) and young adults (center) are relatively normal, but all adults (right) develop clinically overt melanomas within 4-12 months. Genomic DNA was isolated from a melanoma as well as surrounding skin in four of these transgenic animals and RADseq libraries were created from gDNA using either SbfI (0.4% of the genome) or NsiI (9% of the genome). **B**. The efficiency of reads mapping to restriction enzyme cut sites in the genome, along with the depth of coverage and the number of mutations discovered in each melanoma. We found a small number of mutations in 3 melanomas when sampling 0.4% of the genome. For one fish, the same genomic DNA was used for RADseq with NsiI, and shows a much higher number of mutations, given the greater coverage of the genome. **C**. A fate-mapping transgenic line was created to assess the efficiency of RADseq on subpopulations of cells. The mitf-BRAF;p53 model was bred with a fluorescent color “switch” line in which a floxed GFP cassette can be swapped for an mCherry cassette upon Cre-mediated recombination. This cross was injected with a melanocyte-specific mitf-CreERT2 plasmid. Upon endoxifen or tamoxifen treatment, a subset of the melanocytes in the skin as well as within the melanoma switched from GFP to mCherry. Genomic DNA was extracted from either the bulk tumor/skin, or from FACS sorted mCherry+ melanocytes from the tumor/skin pair. **D**. Overall variant allele fractions across all mutations in the sorted *vs*. unsorted populations showed a significant increase in the mCherry+ subpopulations (*,*p* < 0.05, *t*-test). **E**. Individual mutation variant allele fractions are shown, consistently demonstrating increased sensitivity in the sorted population, which facilitates higher-confidence mutation calling in subclonal populations.

We applied RADseq to a zebrafish model of melanoma to identify mutations from matched pairs of tumor and normal tissue (Figure 2A). Transgenic *p53*-deficient zebrafish expressing the mutant form of human *BRAF(V600E)* in melanocytes spontaneously develop melanoma at 4-12 months of age ; *Tg(mitfa:BRAF(V600E)); p53****−/−*** [28, 29]. We dissected melanoma tumors and normal skin from three adult fish and used the restriction enzyme SbfI (a rare cutter, with an 8bp recognition sequence) to prepare RADseq libraries for sequencing on the Illumina platform (HiSeq2500). In silico analysis using the zebrafish reference genome [30] predicts 30,667 SbfI restriction enzyme cut sites in the genome. Because the recognition sequence of the restriction enzyme is palindromic, each occurrence of the recognition sequence results in two cuts, one on each opposing DNA strand. This generates sequence coverage in both directions from the cut site [22, 23]. We sequenced 100bp flanking both sides of each cut site in the 1.4Gb genome, resulting in representation of approximately 0.4% of the genome. The average depth of coverage for these loci was ~350x, and coverage was even across loci (358x mean, 337x median).

After filtering reads for quality, we found that 91.5% of sequencing reads successfully mapped to the zebrafish reference genome and 94.2% of these reads mapped specifically to the predicted cut site regions. This degree of specificity is striking, especially considering the high genetic divergence between different experimental lines of zebrafish, including differences between our lab strains and the sequenced reference line that we used to predict cut sites [31, 32].

#### Mutation-calling in tumors

RADseq data can be analyzed using standard tools for alignment to a reference genome and genotyping/mutation calling [19]. We aligned reads to the zebrafish (danRer7) reference genome using BWA [33] and identified mutations with MuTec [34] (see methods). By comparing melanoma tumor to normal skin for each adult fish, we were able to identify single nucleotide variants (SNVs). The total number of SNVs identified in the three adult fish tumors were 3, 8, and 5 (Figure 2B). These numbers are consistent with previous characterization of engineered zebrafish melanomas from whole exome sequencing which capture ~3% of the genome and find low mutational burden on average, with significant variation among tumors [35].

#### Restriction enzyme selection

To demonstrate how the choice of restriction enzyme influences the number of mutations captured with RADseq, we used the same genomic DNA from one of the SbfI-digested fish described above and made an additional RADseq library using a more frequently-cutting enzyme, NsiI (a 6bp recognition sequence). In silico analysis predicts 687,595 NsiI cut sites in the zebrafish genome, and again we found our reads mapped to these regions with an on-target rate greater than 90%. Sequencing 100bp flanking each side of these cut sites results in representation of approximately 9% of the zebrafish genome. As expected, with many more bases in the genome represented in the NsiI library, the number of mutations was greater than for the SbfI library - we detected 47 mutations in NsiI-flanking regions *versus* the 5 mutations detected in the SbfI-flanking regions of the same melanoma tumor (Figure 2B and Supplemental Table 1).

#### Lineage-tracing model to study tumor evolution of subclonal populations

One of the challenges to studying genetic evolution in tumors is heterogeneity - multiple subclonal tumor lineages typically make up a single biopsy or tissue sample [36]. In addition, normal cells such as fibroblasts and immune cells are present within a dissected tumor sample, at varying frequencies [37]. This means that typical libraries prepared for tumor sequencing represent a mixture of cells with different genotypes. Single-cell sequencing has promise to overcome some of these challenges, but it remains technically difficult and expensive to characterize comprehensive mutational profiles from single cells [38].

We developed a transgenic zebrafish with an inducible system to selectively trace subsets of melanocytes both in normal skin as well as within the melanoma (Figure 2C). We started with an existing zebrafish line (*ubi:Switch*) [39, 40] that possesses a *ubi:loxP- GFP-loxP-mCherry* cassette. These fish ubiquitously express GFP until exposed to Cre recombinase, which induces a deletion of the GFP coding region and expression of mCherry. We crossed these zebrafish with the (p53^−/−;^ mitfa-BRAFV600E ^+/+^) zebrafish line that spontaneously develops melanoma. Into this background, we injected an inducible Cre transgene (*mitfa:Cre****ERt2****-SV40*) under the control of the mitfa melanocyte-specific promoter [41]. Exposure to the drug Tamoxifen induces Cre-mediated excision of the GFP-lox complex in melanocytes specifically, causing those cells to express mCherry, while the rest of the somatic cells continue to express GFP. We screened for fish that successfully integrated the transgene into their germline and whose offspring show a strong and specific switching of GFP to mCherry expression in melanocytes. The result is a stable line of melanoma-prone zebrafish with inducible fluorophore-based lineage tracing in melanocyte lineages. These fish can be treated with tamoxifen as embryos or adults to induce an irreversible color change in melanocytes. After the drug is removed, the labeled cells continue to express mCherry, and they pass this change on to their daughter cells, resulting in labeled clonal lineages that can be separated from other cells using FACS.

We found that the RADseq method can be used to identify mutations in sorted tumor populations from these animals. We applied tamoxifen to fish with melanoma tumors to label melanocytes in tumors and normal skin before dissection. We dissected tissue, disassociated cells, and used FACS to separate the mCherry+ melanocytes from contaminating stromal cells, and prepared the cells for downstream sequence analysis. We also reserved a portion of each dissected tissue for traditional RADseq analysis of the unsorted population. Nine mutations were identified in both the sorted and unsorted analyses. These mutations show a significantly higher frequency in the sorted (M = 30.56) than the unsorted (M = 25.67) population; t(8) = 7.72, *p* < .0001 (Figure 2D/2E and Supplemental Table 2) which is consistent with the expectation that the specific sorting of melanocytes allowed us to reduce the number of contaminating normal cells in the sample. We believe this system will provide a powerful system to isolate and study subclonal populations of normal and tumor melanocytes throughout development.

#### Capturing somatic mosaicism with RADseq

**Figure 3:**
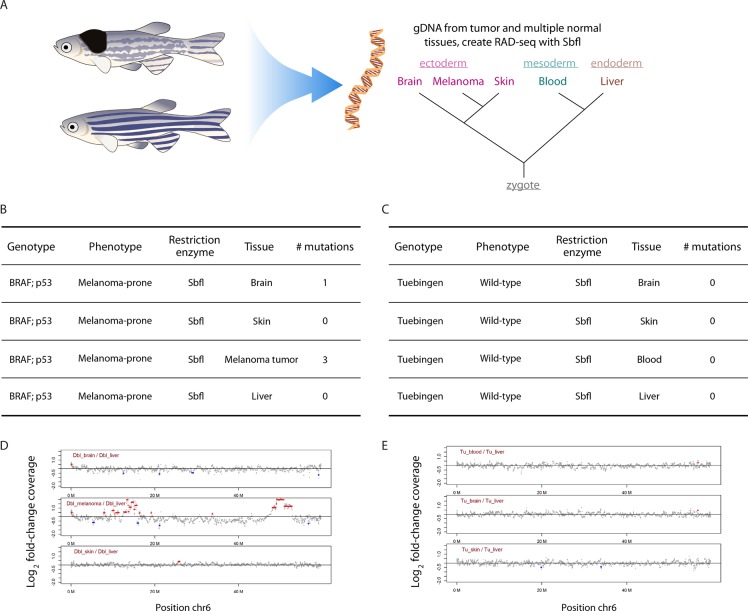
RADseq can identify somatic mosaicism **A**. Multiple normal tissues were extracted from both WT and BRAF;p53 fish, including brain, skin, blood, liver, and spleen. Genomic DNA was isolated and RADseq performed using NsiI. As shown in A., these tissues represent ontologically distinct origins from the zygote. **B**. Mutations were detected in the melanoma as expected. In addition, a somatic mutation was identified and validated in the seemingly-normal brain of the BRAf;p53 fish, an area devoid of BRAF expression. **C**. No somatic mutations were detected in any of the tissues derived from the WT animal. **D**., **E**. The RADseq library was used to detect copy number changes in the above tissues. This revealed two significant copy number amplifications on chromosome 6 in the melanoma, whereas all other tissues from both WT and BRAF;p53 were largely diploid.

We dissected and sequenced different tissues from a melanoma-prone zebrafish and a wild-type zebrafish to test whether acquired genetic variation could be also be detected in somatic lineages other than the tumor population, given that the fish were p53 deficient in all tissues. To do so, we dissected two additional normal tissues, brain and liver, to supplement the skin and melanoma samples from one of the transgenic fish described above. We also isolated brain, skin, blood and liver samples from a wild-type zebrafish. Our selection of skin, blood, and liver was based on the fact that these tissues are derived from different developmental germ layers (ectoderm, mesoderm, and endoderm, respectively) and therefore form distinct lineages early in embryonic development and have the most potential to acquire distinct somatic changes (Figure 3A). The brain is derived from the ectoderm along with skin, and we included this additional tissue because acquired somatic mosaicism has been previously described in this organ [42–45]. We used SbfI to RAD-sequence each of these tissues (see Supplemental Table 3 for RADseq metrics), and detected a somatic mutation specific to the brain sample of the transgenic fish (i.e. a polymorphism present in the brain tissue, but not in the liver, skin, or melanoma tumor from that same animal) (Figure 3B). We confirmed the presence of this mutation in the brain (and absence in other tissues) through targeted PCR of the original genomic DNA and additional deep sequencing of the PCR amplicons to ~1000x coverage in a subsequent HiSeq run. The mutation has a frequency of over 20% in the brain, which indicates that the mutation likely occurred early in development. We did not detect derived mutations in any tissues of the wild-type fish (Figure 3C).

#### Assessing copy number alterations with RADseq

RADseq has been previously shown to be an effective tool to identify copy number alterations in human tumors [46]. To our knowledge, this study by Zheng et al. is the only previous application of the RADseq method to cancer. We analyzed the SbfI-digested RADseq samples from normal and melanoma tissue from melanoma-prone and wild-type fish for evidence of copy number alterations. We identified two regions of chromosome six in the melanoma tumor sample that show strong evidence of amplification (Figure 3D). These regions contain 116 genes, and are not among previously-documented recurrently amplified regions in engineered zebrafish melanomas [35]. Although we cannot evaluate the functional importance of copy number alterations from sequence data alone, the amplified regions do contain several genes of interest including the zebrafish ortholog of the human transcription factor, Myc, which is a frequently mutated oncogene in humans including amplification/overexpression in 6% of melanomas in the TCGA database [47, 48].

We did not detect any copy number changes in any of the normal tissues of the melanoma-prone fish or the wild-type fish (Figure 3E). RADseq has specific advantages for studies of copy number alterations because the selection of restriction enzyme sets the resolution at which copy number change events can be detected. A frequently-cutting enzyme will produce a high density of markers throughout the genome to identify amplifications and deletions of small genomic regions. A rare cutter will produce a lower density of markers spread across the genome, and can be used to identify amplifications and deletions of large genomic regions, with very low sequencing cost.

### Human pancreatic cancer samples from primary and metastatic sites

**Figure 4:**
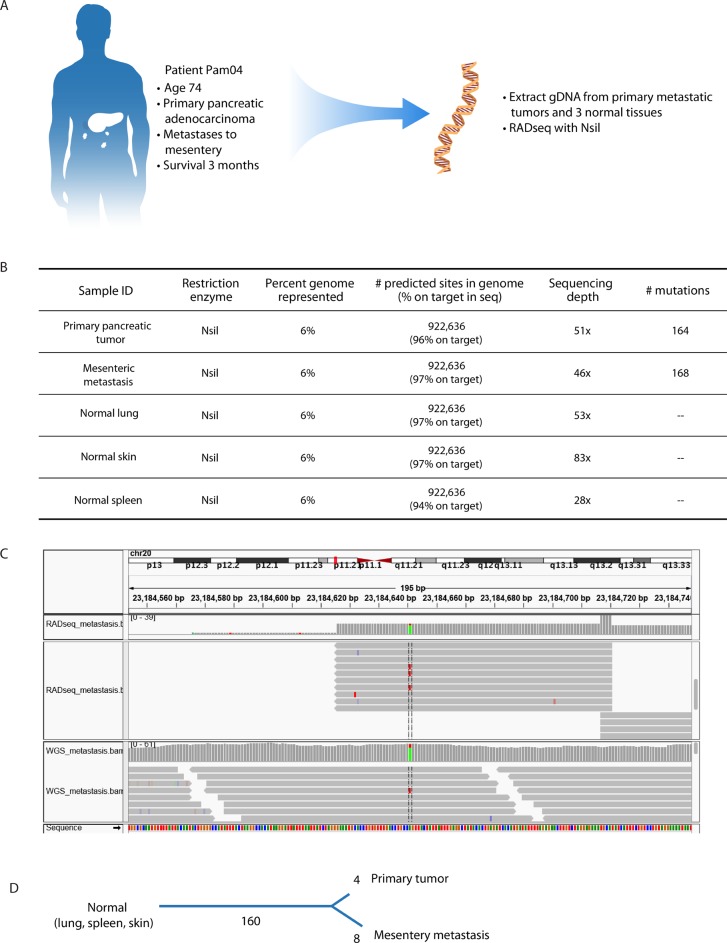
RADseq is adaptable to human cancer specimens **A**. Through a rapid autopsy program, tissues were obtained from a patient with metastatic pancreatic ductal adenocarcinoma (PDAC). These included three tumor-free normal tissues (lung, spleen, kidney), the primary pancreatic tumor, and a mesenteric metastasis. Genomic DNA was isolated from these tissues and subject to RADseq using NsiI. **B**. We identified 168 mutations in the metastasis and 164 mutations in the primary tumor. **C**. An example IGV plot showing the same mutation on chromosome 20 identified by RADseq (top) and whole-genome sequencing (bottom). RADseq shows bidirectional reads emanating from the cut site, in contrast to the random tiling of reads in the whole-genome approach. **D**. Mutations in the primary tumor and metastasis were used to construct a phylogenic tree depicting the evolutionary history among the sequenced populations. 160 mutations are present in the primary tumor and metastasis, indicating that these mutations were already present in the ancestral population that gave rise to the two tumors. Four mutations are unique to the primary tumor, and eight mutations are unique to the metastasis, indicating that these mutations occurred after the two populations diverged.

A unique feature of RADseq is that the same protocol and reagents can be used for any species because there are no sequence-specific capture steps (e.g. exonic baits) involved. We used the enzyme NsiI to generate RADseq libraries for tissue samples obtained from a human pancreatic cancer patient through a rapid autopsy program (Figure 4A) [11]. In silico analysis using the human reference genome hg19 predicts 922,636 NsiI cut sites in the genome. Sequencing 100bp flanking both directions of each cut site results in representation of approximately 6% of the human genome. Note that this is a larger fraction of genome coverage than is targeted by common exome capture kits which typically represent approximately 50-65Mb (~2% of the human genome). Similar to performance in zebrafish described above, RADseq showed very high efficiency with the NsiI enzyme in human tissues (see Supplemental Table 4 for RADseq metrics). After filtering for quality, 92.4% of reads successfully mapped to the human reference genome. 97.0% of these reads mapped to the predicted cut site regions, and 99.28% of predicted cut site regions received sequence coverage. The average depth of coverage was ~50x (52x mean, 49.6x median).

The tissues included in this study were primary pancreatic tumor, mesentery metastasis, and normal spleen. These tissues had already undergone whole-genome sequencing (WGS), and thus provided an independent and comprehensive sequence dataset that we could use to validate RADseq performance for identifying mutations. After filtering for quality, we aligned reads to the reference genome using BWA, and we called mutations with MuTect. We identified 168 mutations distinguishing the metastasis from the normal sample and 164 mutations distinguishing the primary tumor from the normal sample (Figure 4B and Supplemental Table 5 for a list of all mutations detected). 116 (67%) of the 172 total unique mutations that we detected fall in intergenic regions, 53 (31%) are in introns, and 3(2%) are in exons (an example of which is shown in Figure 4C). This distribution is consistent with the composition of the human genome, and supports the prediction that RADseq loci are distributed approximately randomly in the genome and that the majority of mutations detected will be functionally neutral. Of the three exonic mutations, one was silent and two were missense mutations, in codon 12 of KRAS and exon 49 of FBN1. The mutation that we detected in codon 12 of KRAS (G12D) is a common and important driver in pancreatic and other cancers and is concordant with that found by whole genome sequencing of these same samples [49–51]. Although RADseq is not an efficient method to search for driver genes in cancer, there happens by chance to be an NsiI cut site adjacent to this important region, thus cut sites adjacent to hot spots can also identify somatic variants of importance.

To compare results between RADseq and WGS, we independently analyzed sequence data from the whole-genome sequence library. All reads were aligned against the human reference genome, and we used MuTect to identify single nucleotide variants. For efficiency, we restricted mutation calling to regions of the genome adjacent to NsiI cut sites. The whole-genome sequence libraries confirmed the presence of 100% of the RADseq-detected variants.

We used mutation information from primary tumor and metastasis to infer a phylogenetic tree that depicts the evolutionary history underlying tumor progression (Figure 4D). 160 mutations occur before the primary tumor and metastasis lineages diverge. 8 mutations occur along the metastasis lineage, and 4 mutations occur in the primary tumor lineage after its split from the metastasis lineage. These results are consistent with previous studies of pancreatic cancer patients that show a long history of shared mutations among primary tumors and metastases, with fewer unique mutations distinguishing individual tumors within patients [11].

## DISCUSSION

When applied to the appropriate research questions, RADSeq provides an efficient, flexible and cost effective method to utilize the power of next-generation sequencing technologies to gain new insights into the ecological and evolutionary dynamics of cancer. Some additional details regarding study design and analysis will be useful for cancer biologists employing this technique.

Study design: When designing a RADseq experiment, the choice of restriction enzyme will determine the number of loci represented in each sequence library. It is important to have an expectation for the number of cut sites produced by potential restriction enzymes in order to design an experiment that will provide sufficient genetic resolution for the research question, and to anticipate how much sequencing will be required to achieve a given depth of coverage per site. The simplest estimate is that an 8-cutter will cut every 4^8^ = 65,536bp and a 6-cutter will cut every 4^6^ = 4,096bp, but many genomes and enzymes will deviate significantly from this expectation [19]. The calculation can be improved by accounting for the GC content of the cut site and genome under study, however, the most accurate estimates for numbers of cut sites can be obtained through in silico analysis from a published reference genome for the species under study. Computational tools exist to help plan an appropriate sequencing effort for a given target depth by estimating the number of loci expected for a given protocol and genome [52]. The number of bases represented per genome will also be determined by the read length of the sequencing technology (typically up to 150bp reads currently with Illumina, for example). Most questions can be sufficiently addressed with short reads and single-end sequencing, but longer reads can also be obtained by assembling contigs from paired-end sequence reads.

Quality and quantity of starting material: RADseq library preparation protocols have been optimized for high-molecular weight genomic DNA, and are not expected to perform as well with highly degraded DNA such as that obtained from FFPE tissue [53]. In degraded samples, small fragments of starting DNA not adjacent to cut sites may end up in the sequencing library and waste sequencing effort on off-target loci. Also, the mechanical shearing step to produce fragments of optimal size for the sequencing platform works best with relatively large fragments present after enzyme digestion. In addition to high quality DNA, a large quantity of starting DNA is beneficial because it can reduce the number of PCR cycles required for the final step of library prep to reduce PCR bias/duplicates. Early RADseq papers recommend as much as 1ug of starting DNA, but more recent studies have shown successful library prep with as little as 50-100ng DNA per sample [19].

Error: RADseq is subject to many of the same sources of error that challenge all high-throughput sequencing studies of cancer genomics. Type II error or ‘false negative’ mutation calls can occur if a mutation is present at low frequency within the tumor population and sequencing depth is insufficient to resolve the variant. RADseq may be less susceptible to this type of false negative than whole exome sequencing or whole genome sequencing because the cost-effectiveness of RADSeq enables greater sequencing depth per site. False negative mutation calls can also occur when tumors contain genetic heterogeneity that is not captured in a single biopsy. RADseq again may have advantages over WES and WGS with regards to this problem because the more cost-effective approach enables the sequencing of multiple spatially-independent tumor samples (multi-region sequencing) instead of a single biopsy. Type I error or ‘false positive’ mutation calls also need to be considered in all high-throughput sequencing studies of tumors. False positives due to mapping errors of reads to the reference genome can be minimized in RADseq with the same bioinformatics preprocessing tools designed to address these problems in whole genome and whole exome studies, such as GATK indel realignment [54]. We find that mapping errors are easier to identify with RADseq than other sequencing methods because the palindromic nature of the restriction enzyme cut sites should result in two sets of reads independently mapping to the same genomic region - one from each direction of the cut site. False positive mutation calls can also result from PCR errors occurring during the library preparation. These errors need to be addressed differently in RADseq than whole genome or whole exome studies, as we discuss in detail below.

PCR errors and duplicates: Like most next-generation sequencing library preparation protocols, RADseq methods include a PCR step to enrich for fragments that have successfully ligated sequencing adapters. This amplification can potentially lead to erroneous downstream mutation calls, if duplicates that contain PCR errors appear as mutations. The standard method to eliminate PCR duplicates from whole-genome and whole-exome sequence libraries involves removing reads that start and end at the exact same genomic position. This method is not appropriate for RADseq libraries because reads consistently begin at the restriction enzyme cut site. To eliminate PCR duplicates from RADseq data, several alternative methods are available. One option is to use paired-end sequencing (i.e. sequence the randomly-sheared end of the DNA as well as the cut-site end, to remove reads with identical start and end positions). Alternatively, parallel PCR reactions can be performed for each sample and sequenced in separate lanes. Our preferred method is to use multiple barcodes for each individual sample (introduced as a mixture during library prep) and ensure that mutations identified at a particular locus are confirmed by reads containing different barcodes (and thus not the result of PCR error and duplication). The use of multiple barcodes also increases the complexity of the library, which leads to better cluster identification on the Illumina platform. A final way to eliminate PCR duplicates is to eliminate the PCR step of library prep altogether, but this requires higher starting quantities of genomic DNA.

Reagents: The RADseq protocol requires an initial financial investment in specialized barcoded adapters, but adapter sequences are non-proprietary and a single set of oligonucleotides is sufficient for a large number of libraries. Additionally, the same set of adapters can be used for compatible sets of enzymes that leave the same sticky end. For example, the two enzymes used in this study (SbfI and NsiI) leave the same ACGT overhang, so we were able to use a single set of adapter oligos to produce libraries from both restriction enzymes. The same set of barcoded adapter oligos can also be used for different species, as we demonstrate with human and zebrafish.

## CONCLUSIONS

Many studies of evolution in cancer are more limited by the number of individuals or tissues sampled than by the density of markers in the genome, and for these studies RADseq will be an especially useful tool. Like other reduced-representation approaches, RADseq provides advantages over whole genome sequencing, such as greater depth of coverage per locus and the sequencing of higher numbers of samples for a given budget. RADseq also offers an alternative to whole-exome sequencing because it captures more neutrally-evolving sites and thus produces more reliable markers to measure intrinsic mutation rates and to infer phylogenetic relationships than sites under strong positive or purifying selection. Our results demonstrate that RADseq can be an effective tool to identify single-nucleotide variants and copy number alterations in humans and animal models. Our detection of somatic changes in normal tissue of adult zebrafish also indicates that RADseq can be useful for studies of somatic mosaicism in development. The transgenic zebrafish that we created can also be used with RADseq to detect mutations in subclonal lineages.

## EXPERIMENTAL PROCEDURES

### Animal husbandry

All zebrafish were housed in a temperature (28.5C) and light-controlled (14h on, 10h off) room. Fish were housed at a density of 5 fish per liter, and fed 3 times per day using brine shrimp and pelleted zebrafish food. All procedures adhered to IACUC protocol #12-05-008 through Memorial Sloan Kettering Cancer Center, as described previously [55].

### Zebrafish tissue dissection and DNA extraction

Adult zebrafish were anesthetized in 0.2% Tricaine and then euthanized by incubation in ice water for 15 minutes. Tissues were dissected according to the protocol and video published at http://www.jove.com/video/1717/ [56]. Blood was collected according to the protocol and video published at http://www.jove.com/video/3865/ [57]. DNA was extracted from dissected tumors and normal tissue using the DNeasy Blood & Tissue Kit (Qiagen), with an RNAse A treatment step.

### Library preparation

RADseq libraries were prepared from extracted DNA according to the method described by Etter et al [22, 23] using the enzymes SbfI-HF and NsiI (New England Biolabs), with 1μg of DNA starting material. The shearing step was performed on a Bioruptor+ sonication device (Diagenode) with 10 cycles of 30 seconds on, 1 minute off (high setting). Zymo DNA Clean and Concentrator kits (Zymo Research) were used for each clean-up step. The final PCR amplifications were run for 12 cycles. We did not pool samples after the P1 ligation — all samples were barcoded and processed separately until final quantification with a Qubit fluorometer (Thermo Scientific) before being combined proportionately for multiplexed sequencing. Four different barcodes were used for each sample to increase library complexity and assist in eliminating PCR errors in downstream processing.

### Human tissue dissection and library prep

Tissue sample processing. The patient and tissues were collected through the Johns Hopkins Gastrointestinal Cancer Rapid Medical Donation program [58]. Informed consent was obtained. Upon opening the body cavity, the entire primary tumor and remaining normal pancreas were dissected along with each metastasis. All tumor and normal tissues were flash-frozen in liquid nitrogen and stored at -80oC. The primary tumor was sliced (0.5 cm thick) and sectioned into 1×1 cm samples as described previously [11]. Macrodissection of each metastasis removed non-neoplastic tissue.

Genomic DNA extraction and quantification. A phenol and chloroform method was used to extract genomic DNA (gDNA) from each tumor sample followed by LINE assay quantification (i.e. counting long interspersed elements (LINE) using real-time PCR). The LINE forward primer used was 5′-AAAGCCGCTCAACTACATGG-3′ and the reverse primer was 5′-TGCTTTGAATGCGTCCCAGAG-3′. The PCR protocol implemented was 50°C for 2 min, 95°C for 2 min, 40 cycles of 94°C for 10 s, 58°C for 15 s, and 70°C for 30 s, 95°C for 15 s, and 60°C for 30 s. All PCR reactions used Platinum SYBR Green qPCR mastermix (Invitrogen).

Whole genome sequencing and alignment. Sequencing libraries were prepared using standard methods for each gDNA sample. Whole genome sequencing (WGS) utilized an Illumina Hi-Seq 2000 platform for 60x target coverage. Sequencing reads were aligned to the hg19 human reference genome.

### Transgenic zebrafish generation

Melanoma-prone zebrafish *Tg(mitfa:BRAF(V600E)); p53****−/−*** [28] were crossed with the ubi;Switch zebrafish line (*ubi:loxP- GFP-loxP-mCherry*) [39]. A plasmid containing *mitf:Cre****ERt2****;SV40* was created in the pDestTol2CG2 destination vector with the Gateway system [59]. This plasmid (25ng/μl) was injected at the single cell stage using a micropipette along with *Tol2* RNA (20ng/μl) according to previously described protocols [39, 60]. Embryos were treated with 20μm 4-OHT (Sigma) at 50% epiboly. At 24 hours, the drug was refreshed and 0.003% of 1-phenyl-2-thiourea (PTU) was applied to temporarily block pigment production in melanocytes and aid in the visualization of fluorophores [61]. Larvae were imaged at 3dpf to look for color change in melanocytes from GFP to mCherry. Fish that showed strong and specific switching were grown up and then in-crossed to produce an F2 population that was also treated and imaged to confirm incorporation of the transgene in the germline. These fish were grown up and genotyped to select for individuals that carry both homozygous mutations required for the development of melanoma as well as demonstrating the strong and specific switching of melanocytes from GFP to mCherry after tamoxifen treatment. These fish were used to generate a stable line and for the sequencing in this study.

### Tissue digestion and FACS sorting

Adult fish were dissected as described above and the excised tissue was placed in an Eppendorf tube containing 500 μL liberase diluted in DMEM media without serum (final: 0.15U/ml). A mini pestle was used to mechanically dissociate the cells. Tubes were then incubated at 37°C until the cells appeared to be completely dissociated in the solution. 500 μL of DMEM10 media (with serum) was added to stop the liberase activity. The cells were then pelleted by centrifugation at 500 RCF for 5 min in a refrigerated centrifuge. The supernatant was discarded and cells resuspended in PBS buffer. The solution was filtered into a FACS tube through a 40 μm mesh filter, with Dapi (1:100). mCherry positive cells were separated from GFP positive cells on an Aria III FACS machine with gating set by controls from pure populations of zebrafish *in vitro* cell lines. DNA was extracted from sorted cells as described above.

### Sequencing method

All sequencing was performed on an Illumina HiSeq 2500 at the University of Oregon. Single-end reads of 100bp or 150bp were generated, and raw reads were exported for downstream analysis.

### Informatics analysis

Filtering for quality. Each lane of data was processed with STACKS [62, 63], through the process_radtags pipeline, which checks for intact barcodes and cut-sites and sorts reads by barcodes into separate files. The pipeline also filters for quality using a sliding window approach and reads were discarded if the score dropped below 90% probability of being correct. The sliding window was set to 15% of the length of the read. Pre-processing. Reads passing the initial quality filter were pre-processed as described in GATK “best practices” [54, 64, 65] with the exception of the de-duplication step because that step is incompatible with RADseq data (see removing PCR duplicates above). Reads were aligned to the zebrafish (danRer7) or human (hg19) reference genomes using BWA [33]. Mutation calling. Mutation-calling was done using MuTect [34]. Manual curation. The mutation lists generated by MuTect were curated *via* manual inspection of the alignment files at each location using the Integrative Genomics Viewer (IGV) [66]. Mutations in regions with problematic mapping or asymmetrical coverage of RADseq reads on each side of the cut site were omitted.

### Copy-number alteration

Restriction sites were located in the zebrafish genome (danRer7 assembly) *via* in silico digestion of SbfI. Only reads that perfectly matched the location of restriction sites were retained to calculate the coverage of the sites. To depress the fluctuation of read depth, we merged 5 consecutive restriction sites as a unit, and walked along the chromosome *via* a sliding-window to calculate average count read number in each unit. The read depth ratio for each unit of the tested sample was calculated by dividing corresponding value in liver sample of melanoma-prone strain or wild-type strain after normalization of total reads counts. The R package “DNAcopy” was used to segment the chromosome *via* read depth ratio of each tested sample. To resolve the hyper-segmentation, the FastCall algorithm [67] was used to merge neighboring segments with similar copy numbers.

## SUPPLEMENTARY MATERIALS TABLES




