# Supplementary material for: Tumor diversity and evolution revealed through RADseq
Source: Oncotarget. 2017 Jun 3;8(26):41792–805. doi: 10.18632/oncotarget.18355 (PMC5522028; doi:10.18632/oncotarget.18355)
Supplement: Supplementary file 1 [file oncotarget-08-41792-s001.pdf]

Tumor diversity and evolution revealed through RADseq

Supplementary Material

For Supplementray Tables 1, 2 see in Supplementary Files.
